# Application of blended teaching model based on SPOC and TBL in dermatology and venereology

**DOI:** 10.1186/s12909-021-03042-7

**Published:** 2021-12-09

**Authors:** Jinrong Zeng, Liyao Liu, Xiaoliang Tong, Lihua Gao, Lu Zhou, Aiyuan Guo, Lina Tan

**Affiliations:** Department of Dermatology, Third Xiangya Hospital, Central South University, No.138 Tongzipo Rd, Yuelu District, Changsha, 410013 Hunan China

**Keywords:** Dermatology, Venereology, Small private online course, Team-based learning, Clinical medicine, Medical education

## Abstract

**Background:**

In this study, we applied the small private online course (SPOC) and team-based learning (TBL) blended teaching model to dermatology and venereology to ensure a higher quality learning experience for clinical medical students.

**Methods:**

A total of 52 fifth-grade clinical undergraduates from Xiangya School of Medicine of Central South University were randomly divided into an experimental (*n* = 26) and a control group (*n* = 26). In March 2018, we used the SPOC and TBL blended teaching model in the experimental group and explored the effects of innovative teaching in the dermatology and venereology course, compared with the control group receiving the conventional teaching method. We analyzed the two groups’ theoretical assessment scores and questionnaire results to evaluate the efficiency of the new pedagogy.

**Results:**

Students in the experimental group had a better understanding than the control group of the dermatology and venereology content and higher scores on the case analysis questions in the final theoretical examination. The results revealed that the majority of the experimental group students agreed that the novel teaching model blended with SPOC&TBL helped them significantly stimulate motivation and develop their ability in self-directed learning, independent thinking, literature retrieval, presentation board, teamwork, communication, and systematic clinical thinking. The teaching satisfaction survey of the two groups showed that the students’ satisfaction in the experimental group was significantly higher than in the control group (*p* < 0.05).

**Conclusions:**

The SPOC&TBL teaching model is better than the traditional one in enriching students’ professional knowledge and cultivating their comprehensive ability. It can effectively promote educational quality, improve students’ learning effects, and enhance their satisfaction. This method has broad application prospects.

## Background

Small private online courses (SPOCs) were first proposed by Professor Armando Fox [1] at the University of California, Berkeley in 2013. They are based on massive open online courses (MOOCs). The SPOC platform has the same abundant educational resources as a MOOC [2], as well as simpler management procedures and lower administration expenses. SPOCs have developed vigorously worldwide in the last few years, bringing a new look to medical education. The team-based learning (TBL) teaching model was proposed by Michaelsen at Oklahoma University in 2002 [3]. It advocates that students are the core of learning activities, cooperating with the team to complete tasks [4]. It can contribute to improving learning efficiency and enable students to apply basic knowledge to clinical cases. The effectiveness of TBL has been confirmed in the teaching practices of many subjects. By establishing two tracks, online (SPOC platform) and offline (TBL classroom), the new hybrid model realizes the importance of the teaching process and its innovativeness.

Dermatology and venereology, a compulsory course for students in clinical medicine, has unique characteristics. As secondary subjects, they study more than 2,000 diseases with complex etiologies involving immune, genetic, infectious, environmental, and other factors, of which the main clinical manifestations are morphological changes. Many students find it difficult to form a preliminary diagnosis by identifying skin lesions in clinical work, even after theoretical learning and clinical probation. In addition, some hold biased stereotypes about dermatological diseases or care about the infectivity of some diseases. Under the influence of these negative cognitions, students lack initiative and enthusiasm in learning dermatology and venereology. Consequently, there is an urgent need to reform the education methods. In this study, we carried out an innovative trial by introducing the hybrid SPOC and TBL teaching model into dermatology and venereology courses, hoping that the novel program can propel students’ interest in them, raise their learning efficiency, cultivate the ability to solve clinical cases, and help them combine theoretical knowledge with clinical practice.

## Methods

### Participants and study setting

The ethics of this study has been approved by the IRB (Institutional Review Board) at the Third Xiangya Hospital, Central South University, China. A total of 52 undergraduates in the fifth year admitted to Xiangya School of Medicine in 2013 volunteered to be enrolled in the research. The teacher collected in advance the students’ grades, gender, past learning experience, course credits, and knowledge of the new pedagogy and grouped the students using a system of linear equations with a minimisation technique to make the comprehensive strength between the teams equal. They were divided into an experimental (*n* = 26) and a control group (*n* = 26) according to the design of this innovative project. There were 13 men and 13 women in the experimental group, aged between 21 and 24 years, with an average age of 22.9±0.6, and 12 men and 14 women in the control group, aged between 21 to 24 years, with an average age of 23.0±0.4. No significant difference was observed in gender, age, or other information between the two groups (*p* > 0.05). The project lasted for two semesters, from March 2018 to March 2019.

### Educational strategies

The study materials and time setting of the two groups referred to the syllabus of dermatology and venereology. Although the lesson forms were different, the two groups of students learned the same knowledge dots. The students in the control group followed the traditional teaching model, in which students previewed mainly through reading textbooks for 10-15minutes rather than videos before class, and teachers used the PPT in a frontal manner to explain professional knowledge in class. Modules such as clinical cases, lecture discussions, and quizzes & answers would not be introduced into the class. Students attended one 2 hours weekly traditional tutorial. The experimental group adopted a 2 hours weekly hybrid teaching model that combined the SPOC platform and a TBL curriculum. The TBL curriculum included the Individual Readiness Assurance Test (I-RAT), Team Readiness Assurance Test (T-RAT), clinical problem solving activities, and peer evaluation. Although there is no separate appeal and dispute module, students can immediately raise their questions and challenges during each discussion session. The implementation process was as follows: (1) The students in the experimental group were divided into four subgroups, each with six or seven members. Teachers had to comprehensively analyze students’ learning ability, personality characteristics, and other factors for the purpose of grouping them in a balanced way. The members elected a student as the group leader, a person who had a sense of responsibility for the team and who coordinated the team’s task distribution and cooperation. (2) Based on the MOOC course of dermatology and venereology uploaded previously, we established an independent SPOC course aimed at medical students in Central South University that was conducive to completing the education reform of the hybrid model based on the SPOC course. We recorded about 900 minutes of instructional videos with reference to the syllabus of dermatovenereology, including 60 theoretical knowledge points. The duration of each SPOC lecture was about 10-15 minutes, and the theoretical test would not take more than 10 minutes. The students can adjust the video playback speed and determine self-study time independently. And we established the related network platform structure, including scoring standards, teaching courseware, assignments, forum, theoretical examination, and other modules. (3) We published the course description on the Internet before starting the class, requiring students to read and be familiar with it. And we reaffirm the course format and settings on the first day of the offline course. (4) Depending on the prearranged plan, students logged on to the SPOC platform to watch the videos for self-directed study before the TBL curriculum and formed the primary knowledge network [2, 5]. Each instructional video had several multiple-choice questions attached, and students could get the reference answers after submitting their answers. Students could post their questions on the forum to communicate with teachers or other students. (5) On completing the online course, students could access discussion topics and clinical cases for the following week’s lectures. The cases discussed in this class had to cover fundamental theories and key points. Some complex cases were designed to guide them to discuss and communicate, aiming to deepen their understanding. Each participant had to complete the tasks by discussing them collectively, and the group leader subsequently recorded when members studied collectively [6]. (6) Individual Readiness Assurance Test (I-RAT): The personal test that all the students had to complete within the limited time before attending the TBL class consisted of 10 closed-book multiple-choice questions, including the fundamental definition and critical points. The purpose of this pre-class assessment was to estimate the effect of students’ preparation. This section took about 10 minutes. (7) Team Readiness Assurance Test (T-RATs): The questions at this stage covered the critical content of professional knowledge and are more complex than the previous stage. Each group chose one member to present the PPT and explain the team’s consensus on the question, revolving around pre-class discussion topics and clinical cases [7]. Other students could comment and raise questions. This took about 60 minutes. (8) Clinical problem-solving activities: This process encouraged students to use the knowledge learned in the previous teaching stage to solve practical problems. The teacher presented extended questions and comprehensive clinical cases for students to discuss immediately, which focused on fostering their team spirit and encouraging them to discuss and interact until reaching a consensus. If there was a dispute, students could give feedback in time and teachers could organize a discussion between groups. The teacher had to pay attention and provide clues when necessary. Finally, the teacher announced the reference answers in class. This took about 30 minutes. (9) Finally, the teacher collected the answer sheets of all the groups and asked them to evaluate the team members’ performance in the group and their contribution to the outcome [8]. The teacher conducted a systematic evaluation at the end of the course, summarized the key points, corrected the errors, expanded the knowledge points, and commented on the class performance of the groups. This took about 20 minutes.

### Evaluating the effect of teaching model application

#### Theoretical examination

1. At the end of the semester, the teacher prepared the examination according to the syllabus and administered a theoretical exam to the two groups covering five concepts (15), 25 multiple choices (50), and five comprehensive analysis questions (35), and calculated the two groups’ scores. Candidates’ names and other personal information are filled in the column on the left side of the test paper, which would be covered after the papers collecting. Then teachers graded the papers according to the prepared suggested answer.

#### Collect questionnaires about SPOC&TBL pedagogy

A questionnaire survey was conducted among students of the experimental group after the SPOC&TBL course. The questionnaire was adapted from a validated questionnaire designed by Thompson and colleagues, combining the objectives defined by Bloom Taxonomy and the need for clinical teaching [9, 10]. To assess the effectiveness of this new teaching method, eight aspects were investigated, including studying motivation, self-directed learning, independent thinking, literature retrieval ability, presentation skills, teamwork ability, communication skills, and clinical thinking ability [11]. The questionnaire was voluntarily filled out and anonymously handed.

#### Survey on student satisfaction

The students’ satisfaction with the pedagogy of the two groups was evaluated along seven dimensions: pedagogy satisfaction, personal performance satisfaction, improved ability by taking class, valuable educational content, appropriate classroom pace, satisfaction with teachers, and requirements of more SPOC and TBL training. The questionnaire was completed and anonymously handed.

### Statistical analysis

SPSS software (version 22.0) was used to analyze the data. Continuous variables were expressed as (`*x* ± *s*), and two-sample *t*-tests were used for between-group comparisons. Categorical data were expressed as percentages and tested using Pearson chi-square tests. Statistical significance was set at *p* < 0.05.

## Results

### The SPOC&TBL group scored higher on the comprehensive analysis questions

There was no significant difference in the average scores of concepts and multiple choices between the two groups (*p* > 0.05). When comparing the average scores of the comprehensive analysis questions between the two groups, the mean score of the SPOC&TBL group (31.27±1.91) was significantly higher than the mean scores of the control group (29.54±2.89), as displayed in Table 1. Our study confirmed that the SPOC&TBL teaching model had more advantages in cultivating students’ ability to analyze and solve clinical issues.

**Table 1:** Comparison of the test scores between two groups

Groups (SPOC&TBL *n* = 26 Non-SPOC&TBL *n* = 26)	Terminology test scores (`x ± s)	Multiple choice question scores (`x ± s)	Comprehensive analysis question scores (`x ± s)
SPOC&TBL	12.19±1.72	42.23±2.85	31.27±1.91
Non-SPOC&TBL	12.23±2.03	42.15±3.44	29.54±2.89
*t*	−0.074	0.088	2.549
*p*	0.941	0.930	0.014

### SPOC&TBL pedagogy to improve comprehensive ability of students

Table 2 displays eight items regarding the questionnaire results about the effectiveness assessment of SPOC&TBL teaching model. We used the number and percentage of options to describe the students’ attitude. The majority agreed that SPOC&TBL helped them raise their studying motivation, independent learning, independent thinking, document retrieval skills, presentation skills, teamwork ability, communication skills, and clinical thinking, indicating that SPOC&TBL hybrid teaching model yielded satisfactory educational effects in dermatology and venereology, improved students’ comprehensive ability, and helped students better adapt to clinical work.

**Table 2:** Analysis of the capabilities obtained by the SPOC&TBL group

Items	Percentage (%)	
	Yes	No
Studying motivation	69.2	30.8
Self-directed learning skills	61.5	38.5
Thinking independently	73.1	26.9
Document retrieval ability	53.8	46.2
Presentation skills	76.9	23.1
Teamwork ability	80.8	19.2
Communication skills	69.2	30.8
Clinical thinking ability	76.9	23.1

### Results of satisfaction survey in the SPOC&TBL Group

Table 3 displays seven dimensions of the satisfaction survey toward the pedagogies that the two groups received. Overall, the response frequency indicated that significant differences (*p* < 0.05) were observed between the SPOC&TBL and the non-SPOC&TBL group for the following statements: “pedagogy satisfaction”; “personal performance satisfaction”; “improved ability by taking the class”; valuable educational content”; and “appropriate classroom pace”. There was no significant difference was observed in “satisfaction with teachers” and “requirements of more SPOC & TBL training” between the SPOC&TBL group and the non-SPOC&TBL group (*p* > 0.05).

**Table 3:** Evaluation of teaching method satisfaction

Groups (SPOC&TBL *n* = 26 Non-SPOC&TBL *n* = 26)		Agree *n* (%)	Neutral *n* (%)	Disagree *n* (%)	χ^2^	*p*
Pedagogy satisfaction	SPOC&TBL	18(69.2%)	5(19.2%)	3(11.5%)	9.99	0.006
	Non-SPOC&TBL	7(26.9%)	15(57.7%)	4(15.4%)		
Personal performance satisfaction	SPOC&TBL	16(61.5%)	8(30.8%)	2(7.7%)	7.789	0.019
	Non-SPOC&TBL	6(23.1%)	16(61.5%)	4(15.4%)		
Improve ability by taking class	SPOC&TBL	19(73.1%)	6(23.1%)	1(3.8%)	6.803	0.023
	Non-SPOC&TBL	10(38.5%)	15(57.7%)	1(3.8%)		
valuable teaching content	SPOC&TBL	20(76.9%)	6(23.1%)	0(0.0%)	8.951	0.008
	Non-SPOC&TBL	10(38.5%)	12(46.2%)	4(15.4%)		
Appropriate classroom pace	SPOC&TBL	21(80.8%)	5(19.2%)	0(0.0%)	18.502	0.00
	Non-SPOC&TBL	7(26.9%)	10(38.5%)	9(34.6%)		
Satisfaction with teachers	SPOC&TBL	16(61.5%)	7(26.9%)	3(11.5%)	1.946	0.473
	Non-SPOC&TBL	9(42.3%)	11(38.5%)	6(19.2%)		
Requirements of more SPOC & TBL training	SPOC&TBL	13(50.0%)	10(38.5%)	3(11.5%)	0.488	0.851
	Non-SPOC&TBL	15(57.7%)	9(34.6%)	2(7.7%)		

## Discussion

In the conventional teaching model, the teacher deconstructs the disease into several parts and then imparts the findings to the students [12], which helps their comprehension under the pace of the class. However, it is challenging for students to adapt to clinical practice, as accounting for theoretical knowledge in the course has little connection with clinical cases [13]. Moreover, students seldom think and explore independently, so they are prone to boredom and lack motivation. The traditional teaching model has an obvious limitation, and there is a need for an innovative one to exercise students’ ability in multiple aspects to cultivate higher-quality clinical talents. As a novel teaching model, SPOC and TBL are different from conventional teaching ones. The SPOC&TBL pedagogy can fit with the community of inquiry framework (CoI), which has proven effective in exploring online learning and blended learning. According to the CoI model, the realization of the online education community depends on three interdependent components: cognitive presence, social presence and teaching presence [14].

Cognitive presence refers to the degree to which learners construct confirmatory meaning through continuous dialogue and reflection [14]. Students develop critical thinking by spiraling upward in a loop of triggering events, exploration, integrated ideas, and solutions. Many studies have shown that students learn more by solving problems than being provided with standard answers; they attempt to construct interpretation more than they receive hermeneutics [15]. SPOC&TBL is beneficial for stimulating learning motivation and encouraging students to ask questions by introducing diverse and vivid teaching modules. Students exercise critical thinking and deepen their understanding of knowledge by solving problems and exchanging feedback.

Social presence includes emotional expression, open communication and group cohesion [14]. The SPOC&TBL model provides students with opportunities to discuss, synchronously or asynchronously, and encourages teamwork and interactive communication to enhance students’ social presence. There is an independent asynchronous discussion board in the platform of SPOC, where students can ask or answer questions, instead answer specific pre-designed questions, and teachers can also provide direct guidance. Students in the TBL model promote learning outcomes improvement through collaborative learning [16]. In a study conducted by the University of California, Berkeley, students who participated in an algebra discussion after class scored a grade higher. The reasons are as follows: Teamwork allows students to complement each other’s perspectives, analyze and solve problems more comprehensively, and promote the development of critical thinking; There is healthy peer competition in the team, which helps stimulate students’ initiative in learning; Students with better grades in the group can help those with lower degrees in the group; Collaborative learning builds positive interpersonal relationships in teamwork, which contributes to students’ mental health. In addition, The SPOC&TBL model highlights the dominant position of students in teaching practice and enhance group cohesion [17, 18].

In asynchronous SPOC discussions, a robust teaching presence has a significant effect on obtaining higher levels of perception and social cohesion. Teaching presence consists of instructional design, direct instruction, and facilitation of learning [14]. To create a teaching presence, teachers need to break down the physical and psychological distance. Teachers can communicate with students and provide direct guidance in the discussion boards on the SPOC platform, which is a straightforward and effective way to express the sense of teaching presence. Research shows that the teachers’ participation in the discussion promotes students’ communication in the asynchronous platform [19]. And teachers can monitor and analyze the viewing progress of educational videos and the content of discussions, which helps to optimize the instructional design and curriculum settings. In addition, Students and teachers have face-to-face contact in offline TBL courses, allowing students to deepen the impression that the online course teacher is a “real person.” That helps to enhance the immersion and strengthen teaching presence.

In general, the hybrid teaching models of SPOC and TBL have many advantages and broad application prospects. However, they remain little applied in education. The curriculum reform in this study confirmed that the SPOC&TBL teaching model contributed to improving students’ ability to solve clinical issues. Most of the students agreed that the new teaching model helped them by stimulating their motivation to study and develop versatile abilities. If we hope to popularize the SPOC&TBL curriculum in many more areas, there are still issues that need to be addressed.

### Improvement of the quality of the teacher team

Unlike the conventional teaching method, the identity of the teacher has changed in the SPOC&TBL curriculum, which requires professional competence and teaching skills. Teachers received relevant training before starting the SPOC and TBL curriculum. Teachers could update the teaching concepts through training. Teachers learn new teaching theories and put them into use. Teachers could develop and design an education portal and courses using more information technology; Teachers could learn and optimize learning materials to construct a more suitable learning environment. With a dominant role in the traditional curriculum, the teacher controls the structure, content, and pace of the curriculum, which is not the case in the SPOC&TBL hybrid teaching model. In the SPOC platform, the teacher collects and integrates learning resources and materials to facilitate students’ independent online learning [20]. In the TBL curriculum, the teacher encourages students to communicate and discuss, resolve doubts and provides flexible and personalized academic guidance. This healthy interaction contributes to the quality of education. Simultaneously, it also indicates that teachers in the SPOC and TBL curriculum need professional knowledge, substantial clinical experience, and excellent leadership capability.

### Optimize the design of course and platform

The selection and design of educational materials for online and offline courses must be continuously optimized according to the characteristics and needs of students. Ideally, independent studying before attending the course helps students establish a knowledge system sufficient to participate in clinical case discussions. To achieve this goal, we should pay attention to their needs and constantly upgrade the SPOC platform. For example, construct a systematic knowledge network and ensure that SPOC courses do not omit essential knowledge points as far as possible. If necessary, add a review section on the platform to help students memorize the knowledge. Add video content navigation and mark the progress bar in the long video to help locate the content pertinently and quickly. In offline TBL courses, it is essential to determine the difficulty of clinical cases for discussion. If we select too complicated cases, it may be difficult for students to find the correct answer, while if it is simple, it is not easy to attract their attention. Teachers should elaborate on abstract concepts in offline courses to supplement online ones, as this is conducive to the internalization and dialectics of knowledge. Some scholars have advocated a combination of TBL and traditional teaching methods in offline classes [21].

### Pay attention to mental health of students and individualized counseling

Students are used to the conventional teaching model and cannot immediately adapt to the new one. Schools can insert the SPOC&TBL curriculum into the traditional one starting from the freshman year [22]. Students who excel are likely to exert peer pressure to others in the group, some of whom may even gradually lose confidence [23]. In addition, some students do not have enough motivation and seldom participate in group tasks. Therefore, it is vital to balance the relationship between individual work and teamwork. Teachers can make members take turns as team leader and organize regular meetings to report their learning progress. While students majoring in clinical medicine have academic pressure, if the task of the SPOC&TBL course is heavy, the quality of students’ task completion may decline, which also needs to be addressed in practice.

### Improve the management and assessment system

Determining a scientific and systematic method to evaluate students’ performance and learning outcomes has always been challenging. Completing an online course on the SPOC platform relies mainly on their initiative and discipline. The website’s supervision mechanism was relatively weak. Although teachers can communicate with students in the class via the Internet, some students’ online presence is limited to their “online identity,” which indicates that students’ attention may be drawn to other things when their website accounts are online. Online anonymous evaluation of group members can help teachers understand the students’ preparation, their contributions in teamwork, whether to supervise or support team members, whether students listen to the opinions of others openly, and the atmosphere of collaboration. Students adjust their roles in the team and participate in group activities with a more positive attitude through impartial mutual evaluation. However, some students contribute little to the TBL group, but obtain high scores by relying on their teammates’ excellent performance. Sometimes close students may find it difficult to evaluate each other honestly. This implies that we need to reform platform construction and establish a more scientific evaluation system in the future.

## Conclusion

There has been no research on the application of the SPOC&TBL teaching model in dermatology and venereology courses. In this study, we introduced it for the first time. Our study confirmed that the teaching model effectively promoted students’ performance and had a positive impact on their learning motivation and comprehensive quality. However, the SPOC and TBL curricula still need improvement, and the effectiveness needs to be verified in teaching practice over an extended period. Our innovative exploration has provided a new perspective for curriculum reform of clinical medicine, and we believe that its education will have a qualitative leap in the future.

## Data Availability

All data generated or analyzed during this study are included in this article.

## Abbreviations

SPOC: Small private online course
TBL: Team-based learning
I-RAT: Individual Readiness Assurance Test
T-RAT: Team Readiness Assurance Test
CoI: Community of Inquiry
